# The First Complete Chloroplast Genome of *Lycium shawii*: Genomic Architecture, Molecular Phylogenetics and Evolutionary Insights

**DOI:** 10.1002/ece3.72972

**Published:** 2026-01-20

**Authors:** Manal Mohammed Ahmed Asiri, Mohammad Ajmal Ali, Mona Solaiman Alwahibi, Sheikh Sunzid Ahmed, M. Oliur Rahman, Rajesh Mahato, Mohammad Faisal, Soo‐Yong Kim, Joongku Lee

**Affiliations:** ^1^ Department of Botany and Microbiology College of Science, King Saud University Riyadh Saudi Arabia; ^2^ Department of Botany University of Dhaka Dhaka Bangladesh; ^3^ ArrayGen Technologies Private Limited Pune India; ^4^ International Biological Material Research Center Korea Research Institute of Bioscience and Biotechnology Daejeon Republic of Korea; ^5^ Department of Environment and Forest Resources, College of Agricultural Life Science Chungnam National University Daejeon South Korea

**Keywords:** chloroplast genome, hypervariable barcodes, *Lycium shawii*, molecular phylogenetics, nucleotide diversity

## Abstract

*Lycium shawii* Roem. & Schult., a stress‐resilient medicinal plant native to the deserts of Saudi Arabia, possesses notable bioactive compounds used in traditional medicine for treating inflammation, oxidative stress, and other ailments. However, its chloroplast (Cp) genome has not previously been sequenced or described, limiting accurate molecular identification and phylogenetic resolution. In this study, we present the first complete Cp genome of *L. shawii* . The plastome spans 155,936 bp and is organized into a large single‐copy (LSC) region (86,608 bp), a small single‐copy (SSC) region (18,430 bp), and two inverted repeats regions (IRA and IRB), each spanning 25,449 bp. A total of 128 genes were annotated, comprising 84 protein‐coding genes, 36 tRNAs, and eight rRNAs. Comparative genomic analyses revealed conservation of genome structure without major rearrangements across Solanaceae. Forty simple sequence repeats (SSRs) and 50 oligonucleotide repeats were identified, with mononucleotides (38) as the dominant SSR type, and forward repeats as the most common among longer repeats. RNA‐editing sites were unevenly distributed, with the highest proportion in the LSC region (48%), followed by the SSC (29%) and IRs (23%). Nucleotide diversity analysis highlighted *atpI*, *rbcL*, and *accD* within the LSC region as hypervariable loci suitable for DNA barcoding. Plastome‐wide phylogenetic reconstruction confirmed the placement of *L. shawii* within the tribe Lycieae of the subfamily Solanoideae. Molecular dating analysis suggested its emergence around 1.40 MYA, during the Calabrian stage of the Cenozoic era. This study provides the first Cp genome resource for *L. shawii* , offering new perspectives for evolutionary and comparative genomics within Solanaceae.

## Graphical abstract

The study presents the first complete chloroplast genome of Lycium shawii (PQ824997.1) spanning 155,936 bp and contains 128 genes (84 protein‐coding genes, 36 tRNAs, and eight rRNAs). Nucleotide diversity analysis highlighted atpI, rbcL, and accD as hypervariable loci suitable for DNA barcoding. Molecular dating analysis revealed that *L. shawii* originated around 1.40 MYA, during the Calabrian stage of the Cenozoic era.

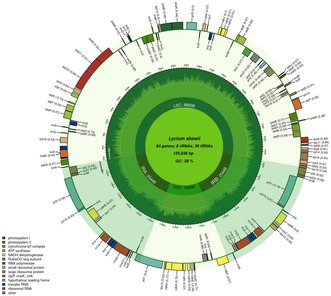

## 1 Introduction

*Lycium shawii* Roem. & Schult. (Solanaceae) is a thorny xerophytic shrub of ecological and medicinal importance, native to the arid and semi‐arid regions of the Arabian Peninsula, especially along sandy and stony ridges in Saudi Arabia (Ahmed and Al‐Dousari 2017). The species is morphologically characterized by sharp thorns, elliptic leaves arranged in congested fascicles, and infundibuliform white or purple corollas. Its flowering period extends from March to April, although continuous blooming may occur under irrigated conditions. The fruit is a globose, many‐seeded berry, red to orange in color, edible and mildly sweet (Suleiman et al. 2011). Ecologically, *L. shawii* plays a vital role in desert habitats, serving as a nectar source for wild Apidae and providing nourishment and shelter for birds and small mammals (Galetto et al. 1998).

*L. shawii* has demonstrated considerable therapeutic potential in multiple pharmacological investigations. Leaf extracts of the species exhibit cytotoxic, antioxidant, antimicrobial, and antiviral properties (Hassan and Abdallah 2017). Methanolic and fractionated extracts have shown cytotoxic effects against various cancer cell lines (HEK293, A‐549, HepG‐2, and MCF‐7), along with notable anti‐inflammatory activity as evidenced by NF‐κB‐luciferase assays. Phytochemical analyses identified diverse bioactive compounds, including phenolics, flavonoids, flavonoid glycosides, and sterols such as luteolin, kaempferol, rutin, and β‐sitosterol (Alkuwari et al. 2012). Antioxidant assays revealed potent radical scavenging activity, while antimicrobial evaluations showed strong inhibition against pathogens, notably *Escherichia coli* . Additionally, in vivo toxicological assessments confirmed the safety of its leaf extracts, reinforcing their potential for pharmaceutical development (Ali et al. 2020).

The chloroplast (Cp) genome is a powerful tool for taxonomic identification owing to its conserved structure, uniparental mode of inheritance, and slower evolutionary rate compared to nuclear genomes, making it particularly valuable for plant phylogenetic studies (Dobrogojski et al. 2020). Conventional DNA barcoding approaches typically target regions such as *trnH‐psbA*, *matK*, *rbcL*, and *ndhF*, which are widely used in plant systematics and are often effective for species‐level identification (Li et al. 2015). However, these markers may lack the resolution needed to distinguish closely related or morphologically similar species. In contrast, whole plastome sequencing, encompassing all protein‐coding genes, tRNAs, and rRNAs, provides a more comprehensive genetic resource, significantly improving species discrimination and phylogenetic resolution (Claude et al. 2025). This genome‐wide approach enables the detection of cryptic species, clarification of ambiguous taxonomic relationships, and deeper insights into evolutionary histories that might be overlooked when using partial sequences (Ahmed and Rahman 2025). Moreover, plastome‐wide molecular dating enhances the precision of divergence time estimates by providing stronger calibration points and reducing uncertainties in molecular clock models. Such integrative analyses contribute to a better understanding of lineage diversification, biogeographic patterns, and evolutionary events (Zhang et al. 2021). Hence, the complete plastome sequence offers a robust molecular basis for the precise identification and phylogenetic placement of *L. shawii* within the Solanaceae family.

Despite its ecological and medicinal importance, the complete plastome of *L. shawii* has remained unexplored, resulting in significant gaps in understanding of its phylogenetic position and evolutionary trajectory through geological time. This study presents the first comprehensive assembly and characterization of the full plastome of *L. shawii* , integrating robust phylogenetic reconstruction with molecular dating analyses. Through this study, we aim to refine its taxonomic resolution, unravel its evolutionary lineage, and provide insights essential for its conservation. Furthermore, this work establishes a foundational genomic resource that will support future research into the ecological, evolutionary, and biological significance of this desert medicinal species.

## 2 Materials and Methods

### 2.1 Sample Collection, DNA Isolation, and Sequencing

**FIGURE 1:**
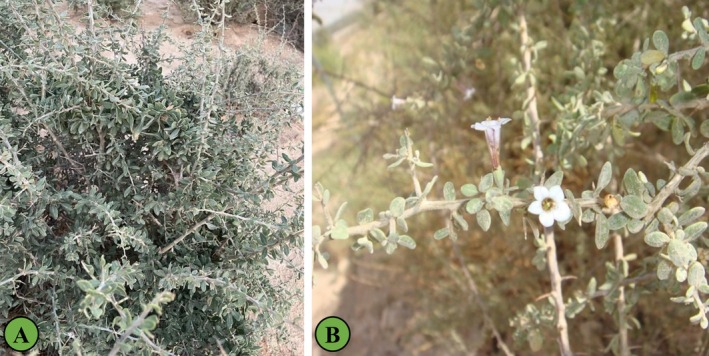
*Lycium shawii* from the Wadi Laban of Saudi Arabia. (A) Habit and (B) flowering twig.

*Lycium shawii*, a species adapted to harsh desert climates, was collected from Wadi Laban, Saudi Arabia (24°36′02.2′′ N, 46°31′59.9′′ E; altitude: 690 m) [Voucher: Asiri, M.M.A. & Ali, M.A. 2023‐1 (KSUH)] (Figure 1). Immediately after collection, fresh leaf tissues were transported to the laboratory and maintained at 4°C under controlled conditions to ensure sample quality. The collected material was subsequently dried with silica gel and preserved at −80°C prior to de novo genome sequencing. Voucher specimens were stored in the King Saud University Herbarium (KSUH), and the taxonomic identity of the plant was authenticated with reference to the Flora of Saudi Arabia (Chaudhary 2001).

Genomic DNA was extracted from the preserved leaf tissues using the Qiagen DNA extraction kit (QIAGEN Inc., UK). Sequencing libraries were constructed to generate 151 bp paired‐end reads on the Illumina NovaSeq 6000 platform (Macrogen, South Korea). The quality of the resulting NGS reads was evaluated employing FASTQC v.0.12.1 to assess quality scores (Ahmed and Rahman 2024; Ali et al. 2024). The raw reads have been stored in the NCBI database under accession number SRR31836791.

### 2.2 Assembly and Annotation

*L. shawii* Illumina reads were assembled using GetOrganelle v.1.7.7.0 to generate the complete plastome. To validate the assembly, raw reads were remapped to the plastome sequence employing the BWA‐MEM algorithm implemented in UGENE v.49.1 to assess coverage depth (Okonechnikov et al. 2012; Jin et al. 2020). Gene annotation was performed with the CPGAVAS2 pipeline and subsequently verified through the CPGView server. The circular plastome map was generated using the Chloroplot server (Shi et al. 2019; Zheng et al. 2020; Liu et al. 2023). The assembled genome was submitted to NCBI with accession ID PQ824997.1.

### 2.3 Repetitive Sequences and Codons

Simple sequence repeats (SSRs) were identified using the MISA‐Web server (Beier et al. 2017) with default parameters, while long repeat sequences were detected through the REPuter server (Kurtz et al. 2001), considering all orientations. Codon usage patterns were analyzed with MEGA v.11 (Tamura et al. 2021). Python libraries, including matplotlib, seaborn, and pandas, were used to visualize codon usage data as a heatmap.

### 2.4 RNA Editing Sites Analysis

RNA editing sites within the plastome were predicted using the PREPACT 3.0 server, applying the BLASTx module to identify forward (C → U) editing events (Lenz et al. 2018). *Nicotiana tabacum* L. belonging to the family Solanaceae was used as the reference genome, with the e‐value threshold set at 0.001.

### 2.5 IR Expansion and Contraction

Structural variation in the inverted repeat (IR) regions of *L. shawii* was analyzed using the IRscope platform (Amiryousefi et al. 2018). The GBF file of *L. shawii* was compared with annotation files from related taxa to examine junction boundaries. The generated visualization plots were then downloaded and inspected to assess IR boundary shifts.

### 2.6 Comparative Plastomics and Nucleotide Diversity

Comparative assessment of the plastome was performed with the mVISTA server (Frazer et al. 2004) to assess genome divergence and conservation among related taxa. The plastomes of *L. shawii* and related taxa were aligned using MAFFT. Nucleotide diversity was estimated from the aligned sequences employing DnaSP v.5 to assess sequence variation across the plastomes (Librado and Rozas 2009; Katoh and Standley 2013).

### 2.7 Phylogenetic Analysis

Complete chloroplast genome sequences were aligned using MAFFT v.7 under the FFT‐NS‐1 algorithm, suitable for large‐scale nucleotide datasets with moderate sequence divergence (Katoh and Standley 2013). Sequence similarity was evaluated using the BLOSUM62 scoring matrix, with a gap‐opening penalty of 1.53 and an offset value of 0, allowing a balanced treatment of insertions and deletions. To improve homology inference and alignment accuracy, particularly across conserved coding regions among divergent lineages, the MAFFT‐homologs option was applied, incorporating up to 600 homologous sequences from the UniRef50 database. This approach enhances alignment reliability by anchoring conserved regions to well‐characterized reference homologs. Ambiguously aligned and highly variable regions were removed using trimAl v.1.4.1 with an automated heuristic strategy, minimizing alignment artifacts and reducing the impact of spurious homology, an important consideration when resolving deep phylogenetic relationships and rapidly radiating lineages (Capella‐Gutiérrez et al. 2009). The final alignment was visually inspected to verify positional homology and subsequently used for all phylogenetic and molecular dating analyses.

Phylogenetic reconstruction was carried out using maximum likelihood (ML) and Bayesian inference (BI) frameworks, both of which are well‐suited for resolving phylogenetic relationships across deep and recent divergences while minimizing artifacts such as long‐branch attraction. ML analyses were conducted using IQ‐TREE v.2.4.0, with the best‐fit nucleotide substitution model selected via ModelFinder Plus (MFP). Model selection was evaluated using the Akaike Information Criterion (AIC), corrected AIC (AICc), and Bayesian Information Criterion (BIC). Based on BIC, TVM + F + R3 was identified as the best‐fitting model and applied for ML tree inference. Branch support for the ML topology was assessed using the ultrafast bootstrap approximation (UFBoot) with 5000 replicates, providing a computationally efficient and statistically robust estimate of nodal support (Minh et al. 2020).

To further evaluate topological stability and account for phylogenetic uncertainty, Bayesian inference was performed using MrBayes v.3.2.7 under the GTR + I + Γ substitution model, which approximates the best‐fit ML model while accommodating among‐site rate heterogeneity and invariant sites (Ronquist and Huelsenbeck 2003). Bayesian analyses comprised two independent Markov chain Monte Carlo (MCMC) runs of 200 million generations with sampling at 10,000‐generations intervals. Convergence and adequate sampling were assessed by examining the standard deviation of split frequencies and ensuring that effective sample sizes (ESS) exceeded 200 for all estimated parameters, indicating sufficient mixing and reliable posterior estimates. The first 25% of sampled trees were excluded as burn‐in prior to reconstruction of the majority‐rule consensus tree. Only nodes that were consistently recovered with strong support in both ML (UFBoot) and BI (posterior probability) analyses were considered robust and retained for downstream interpretation, thereby reducing the influence of potential phylogenetic artifacts.

### 2.8 Molecular Dating Analysis

Divergence times were estimated using the RelTime‐ML method implemented in MEGA v.11, which estimates node ages without assuming a strict molecular clock while allowing rate variation among lineages, making it suitable for datasets encompassing both deep and shallow divergences, such as complete chloroplast genomes (Tamura et al. 2021). This approach was applied following the protocol of Mello (2018) and has been widely used in recent phylogenomic studies to reliably estimate divergence times in plants and other taxa (Garcia‐R et al. 2020; Lin et al. 2021; Chen et al. 2022; Zhu et al. 2025), demonstrating its methodological appropriateness for our study. Two secondary calibration points from the TimeTree database were applied with uniform priors: (i) the divergence between *Nicandra physalodes* and *Atropa belladonna* (11.2–45.9 million years ago) and (ii) the divergence between *Atropa belladonna* and *Lycium shawii* (0.0–16.9 million years ago) (Kumar et al. 2017). The GTR substitution model was used, with rates among sites modeled using a gamma distribution with five discrete categories. Sites with gaps or missing data were treated using partial deletion with a 95% site coverage cutoff. Divergence times were inferred from the resulting time‐calibrated tree, providing a robust temporal framework for interpreting rapid radiation events within *Lycium*.

## 3 Results

### 3.1 Assembled and Annotated Plastome

Whole genome sequencing (WGS) of *L. shawii* was carried out using the Illumina NovaSeq 6000 platform, yielding 404,523,417 raw reads (spots), comprising approximately 122.2 gigabases of sequence data with an estimated data size of 36.5 GB (SRR31836791). Quality assessment with FASTQC revealed GC contents of approximately 38% and 39% for the forward and reverse reads, respectively, with a sequence length of 151 bp. No sequences were flagged as low quality. Per‐base sequence quality of paired‐end reads demonstrated consistently high‐quality scores across the entire read lengths. Forward reads exhibited very good quality, with mean Phred scores exceeding 38, and median, lower quartile, and upper quartile values uniformly at 40, indicating minimal variation and superior base‐calling accuracy throughout, including at the 3′ end. Reverse reads also passed all quality checks, with median and quartile values remaining at 40, though a slight decline in mean scores was observed toward the end, decreasing from above 38.4 in the early bases to approximately 36.3 at base 151. Despite this minor drop, the 10th percentile values remained above 24, confirming that both read directions were of high integrity and suitable for robust genome assembly and downstream analyses.

**FIGURE 2:**
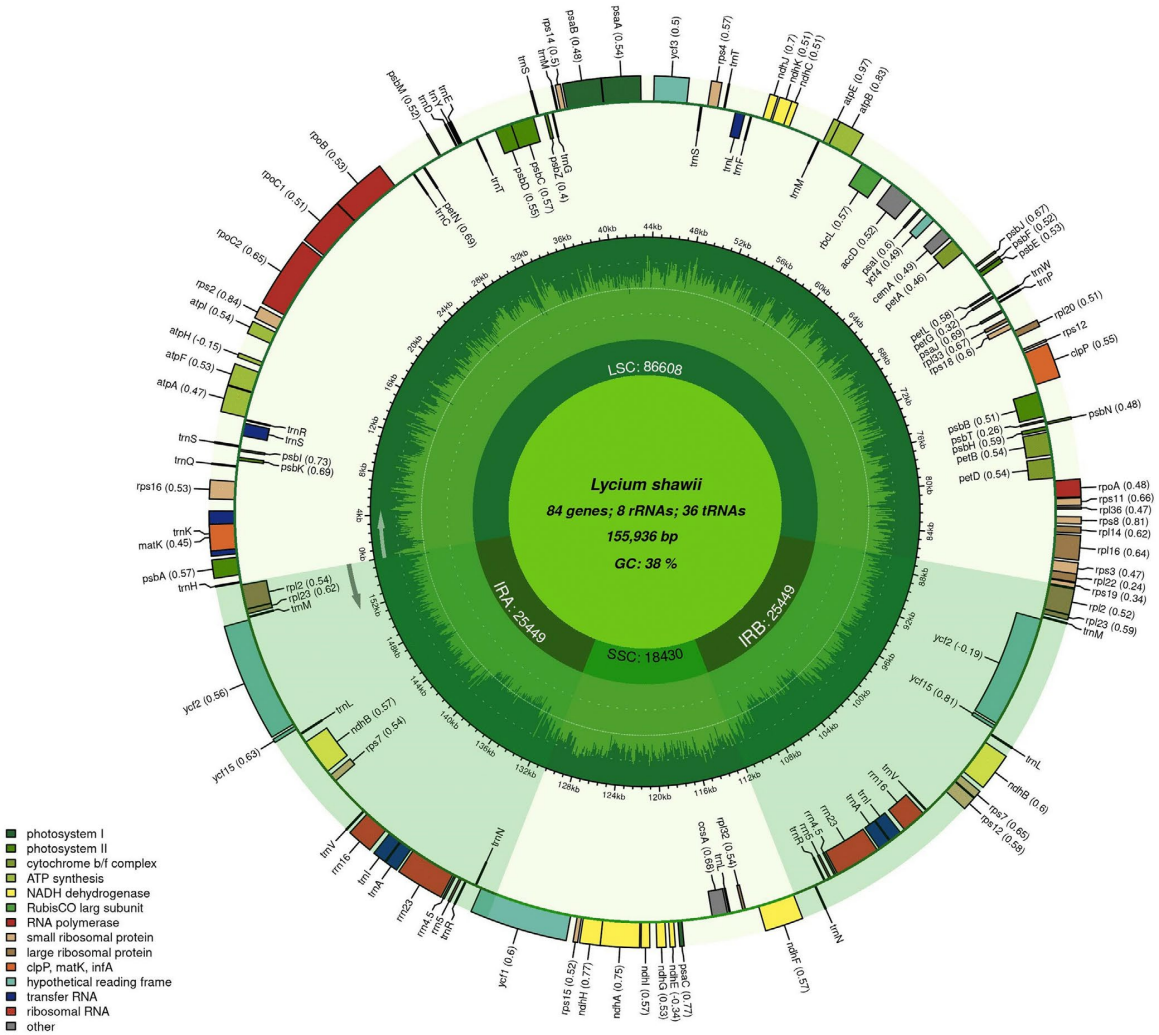
Orbicular plastome map of *L. shawii* elucidating the arrangements of protein‐coding genes, transfer RNAs, and ribosomal RNAs.

Assembly with the GetOrganelle tool yielded a circular plastome in *L. shawii* spanning 155,936 bp in length, exhibiting the typical quadripartite structure (Figure 2). The plastome comprised a large single‐copy (LSC) region of 86,608 bp, a small single‐copy (SSC) region of 18,430 bp, and two inverted repeat (IR) regions (IRA and IRB), each spanning 25,449 bp.

Nucleotide composition analysis revealed distinct regional variation in base proportions across the plastome, reflecting functional and structural differentiation among its components. The plastome of *L. shawii* exhibited a high AT content of 30.67% adenine (A) and 31.48% thymine (T), resulting in a total AT content of 62.15%. Cytosine (C) and guanine (G) contributed 19.24% and 18.61%, respectively, yielding a GC content of 37.85% (Table 1). Among the genomic regions, the SSC region was the most AT‐rich (67.65%), followed by the LSC region (64.10%), whereas the IR regions (IRA and IRB) showed higher GC content (43.17%) and correspondingly lower AT content (56.83%).

**TABLE 1:** Nucleotide arrangements in the plastome of *Lycium shawii*.

Area	Cytosine (%)	Guanine (%)	Adenine (%)	Thymine (uracil) (%)	Cytosine + guanine (%)	Adenine + thymine (%)
Small‐single copy	16.86	15.49	33.68	33.97	32.35	67.65
Large‐single copy	18.37	17.53	31.35	32.75	35.90	64.10
Inverted repeat A	20.74	22.43	28.49	28.34	43.17	56.83
Inverted repeat B	22.43	20.74	28.34	28.49	43.17	56.83
Plastome	19.24	18.61	30.67	31.48	37.85	62.15

**FIGURE 3:**
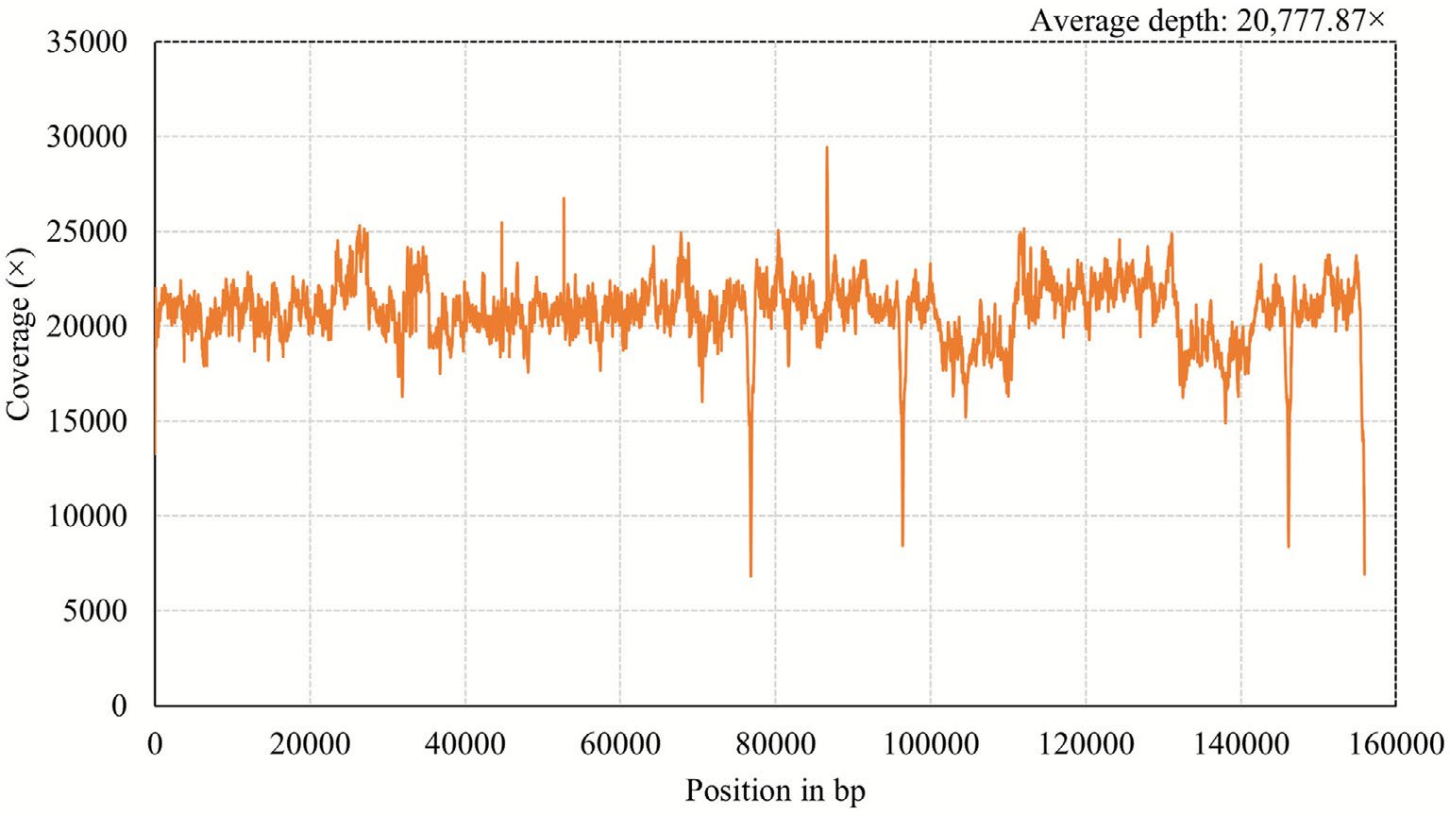
Coverage depth analysis of the assembled plastome of *L. shawii* .

Coverage assessment of the *L. shawii* plastome unveiled high sequencing depth across the genome, confirming the accuracy and completeness of the assembly. The highest coverage depth was 29,441× at position 86,639 bp, whereas the lowest coverage depth was 6812× at position 76,819 bp (Figure 3). The average coverage depth reached 20,777.87×, indicating extensive sequencing redundancy. This exceptionally high coverage ensures that the assembled chloroplast genome sequence is accurate and free from gaps, with minimal risk of base‐calling errors or misassemblies.

A total of 128 genes were annotated in the plastome of *L. shawii* , including 84 protein‐coding genes (PCGs), 34 transfer RNA (tRNA) genes, and eight ribosomal RNA (rRNA) genes (Table 2). Photosynthesis‐related genes comprised six ATP synthase subunit genes (*atpA*, *atpB*, *atpE*, *atpF*, *atpH*, and *atpI*), 14 genes encoding photosystem II subunits, 11 NADH‐dehydrogenase subunit genes (including a duplicated *ndhB*), six genes of the cytochrome *b*/*f* complex, five photosystem I subunit genes, and one gene (*rbcL*) encoding the rubisco large subunit. The self‐replication category comprises genes for the large and small subunits of the ribosome (e.g., *rpl* and *rps* genes, with duplications in *rpl2*, *rpl23*, *rps7*, and *rps12*) and four RNA polymerase subunit genes (*rpoA*, *rpoB*, *rpoC1*, and *rpoC2*). Additionally, the plastome encodes other functional genes such as *accD*, *ccsA*, *cemA*, *clpP*, and *matK*. Several conserved hypothetical open reading frames (*ycf1*, *ycf2*, *ycf3*, *ycf4*, and *ycf15*) were also identified, representing genes of unknown function.

**TABLE 2:** Protein‐coding genes present in the plastome of *Lycium shawii*.

Name of genes	Group	Category
*atpA*, *atpB*, *atpE*, *atpF*, *atpH*, *atpI*	Subunits of ATP synthase	Genes for photosynthesis
*psbA*, *psbB*, *psbC*, *psbD*, *psbE*, *psbF*, *psbH*, *psbI*, *psbJ*, *psbK*, *psbM*, *psbN*, *psbT*, *psbZ*	Subunits of photosystem II	
*ndhA*, *ndhB*(×2), *ndhC*, *ndhE*, *ndhF*, *ndhG*, *ndhH*, *ndhI*, *ndhJ*, *ndhK*	Subunits of NADH‐dehydrogenase	
*petA*, *petB*, *petD*, *petG*, *petL*, *petN*	Subunits of cytochrome b/f complex	
*psaA*, *psaB*, *psaC*, *psaI*, *psaJ*	Subunits of photosystem I	
*rbcL*	Subunit of rubisco	
*rpl2*(×2), *rpl14*, *rpl16*, *rpl20*, *rpl22*, *rpl23*(×2), *rpl32*, *rpl33*, *rpl36*	Large subunit of ribosome	Self‐replication
*rpoA*, *rpoB*, *rpoC1*, *rpoC2*	DNA dependent RNA polymerase	
*rps2*, *rps3*, *rps4*, *rps7*(×2), *rps8*, *rps11*, *rps12*(×2), *rps14*, *rps15*, *rps16*, *rps18*, *rps19*	Small subunit of ribosome	
*accD*	Subunit of Acetyl‐CoA‐carboxylase	Other genes
ccsA	c‐type 21ytochrome synthesis gene	
*cemA*	Envelop membrane protein	
*clpP*	Protease	
*matK*	Maturase	
*ycf1*, *ycf2*(×2), *ycf3*, *ycf4*, *ycf15*(×2)	Conserved open reading frames	Unknown

Exon‐intron analysis revealed that most intron‐containing genes were located mostly in the LSC region, followed by the IRs and the SSC zone (Table 3). Among the 86 PCGs, 72 exhibited no introns. Both *ycf3* and *clpP* contained two introns and three exons. Four distinct tRNA genes—*trnK‐UUU*, *trnS‐CGA*, *trnL‐UAA*, and *trnA‐UGC* harbored a single intron located between two exons. Among the conserved open reading frames, only *ycf1* and *ycf3* demonstrated more than one exon.

**TABLE 3:** Distributions of exons and introns in the plastome of *Lycium shawii*.

Location	Gene	Strand	Start	End	ExonI	IntronI	ExonII	IntronII	ExonIII
LSC	*trnK‐UUU*	−	1794	4376	37	2510	36		
LSC	*rps16*	−	5067	6155	40	822	227		
LSC	*trnS‐CGA*	+	8966	9732	31	676	60		
LSC	*atpF*	−	11,676	12,934	145	704	410		
LSC	*rpoC1*	−	21,049	23,831	432	737	1614		
LSC	*ycf3*	−	43,832	45,835	124	739	232	758	151
LSC	*trnL‐UAA*	+	48,830	49,411	35	497	50		
LSC	*clpP*	−	72,460	74,493	71	805	294	638	226
LSC	*petB*	+	77,440	78,830	6	743	642		
LSC	*petD*	+	79,021	80,245	8	742	475		
LSC	*rpl16*	−	83,648	85,068	9	1016	396		
LSC	*rpl2*	−	86,726	88,216	391	666	434		
IR	*ndhB*	−	97,077	99,288	775	679	758		
IR	*trnA‐UGC*	+	105,452	106,205	37	681	36		
SSC	*ndhA*	−	121,605	123,852	553	1156	539		
SSC + IR	*ycf1*	−	125,807	131,482	1797	42	3837		
IR	*trnA‐UGC*	−	136,340	137,093	37	681	36		
IR	*ndhB*	+	143,257	145,468	775	679	758		
IR	*rpl2*	+	154,329	155,819	391	666	434		

### 3.2 Repeats and Codons

**FIGURE 4:**
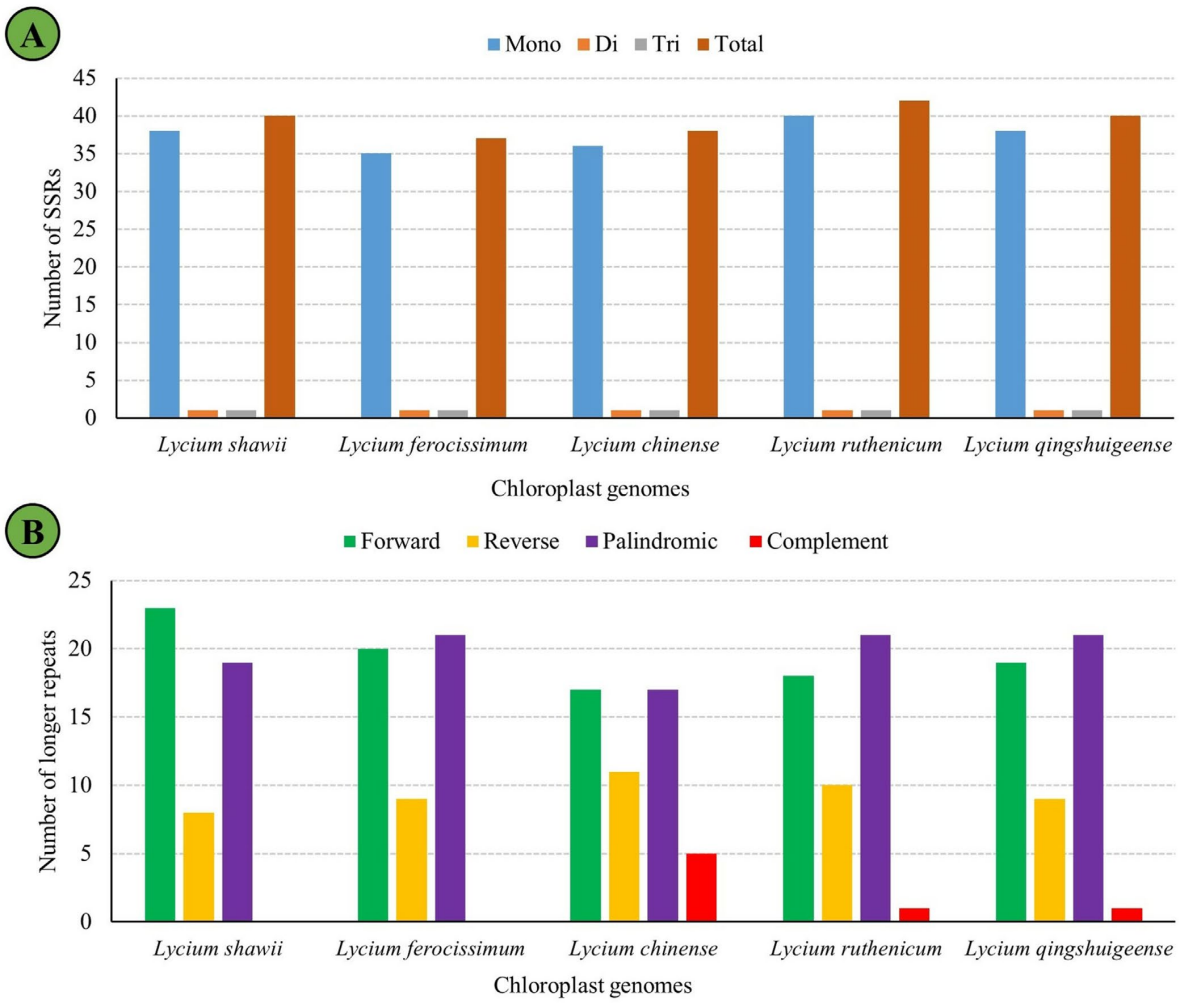
Evaluation of repeat structures present in the Cp genome of *L. shawii* and other closely related taxa. (A) Ditribution of simple sequence repeats (SSRs) and (B) distribution of longer repeats.

Repeat analysis identified 40 SSRs and 50 oligonucleotide repeat structures in the *L. shawii* plastome (Figure 4). Among the SSRs, mononucleotides (38) were the most abundant, followed by di‐ and tri‐nucleotide repeats (Table 4). The distribution of SSRs in *L. shawii* was largely similar to that observed in other *Lycium* species (Figure 4A). Analysis of longer repeat structures revealed variation in repeat types among the species (Figure 4B). In *L. shawii* , 23 forward, 8 reverse, and 19 palindromic repeats were detected, with no complementary repeats identified. Across the five *Lycium* species, forward and palindromic repeats were the most abundant, while complementary repeats were rare or absent. Similar to *L. shawii* , *L. ferocissimum* lacked complementary repeats entirely. In contrast, *L. chinense* showed a notably higher number of complementary repeats (5) compared with the other species. *L. ruthenicum* and *L. qingshuigeense* exhibited similar repeat profiles, each containing only a single complementary repeat.

**TABLE 4:** Simple sequence repeats in the Cp genome of *Lycium shawii* with locations and size distribution.

Sl. no.	SSR type	SSR	Size	Starting position (bp)	Ending position (bp)
1	Mononucleotide	(T)12	12	3772	3783
2	Mononucleotide	(A)10	10	4599	4608
3	Dinucleotide	(AT)6	12	6354	6365
4	Mononucleotide	(T)14	14	6688	6701
5	Mononucleotide	(T)14	14	9543	9556
6	Mononucleotide	(A)12	12	9879	9890
7	Mononucleotide	(T)11	11	9992	10,002
8	Mononucleotide	(T)14	14	12,353	12,366
9	Mononucleotide	(A)12	12	16,529	16,540
10	Mononucleotide	(T)10	10	18,755	18,764
11	Mononucleotide	(A)10	10	29,795	29,804
12	Mononucleotide	(T)10	10	31,137	31,146
13	Mononucleotide	(T)10	10	33,165	33,174
14	Mononucleotide	(A)10	10	33,653	33,662
15	Mononucleotide	(T)12	12	36,557	36,568
16	Mononucleotide	(T)15	15	44,703	44,717
17	Mononucleotide	(A)11	11	45,687	45,697
18	Mononucleotide	(T)10	10	46,233	46,242
19	Trinucleotide	(TTA)5	15	52,715	52,729
20	Mononucleotide	(T)10	10	56,267	56,276
21	Mononucleotide	(A)12	12	56,905	56,916
22	Mononucleotide	(T)10	10	59,070	59,079
23	Mononucleotide	(T)10	10	65,521	65,530
24	Mononucleotide	(A)10	10	67,661	67,670
25	Mononucleotide	(T)10	10	68,484	68,493
26	Mononucleotide	(T)10	10	69,347	69,356
27	Mononucleotide	(T)14	14	71,547	71,560
28	Mononucleotide	(T)10	10	72,084	72,093
29	Mononucleotide	(T)15	15	73,036	73,050
30	Mononucleotide	(A)10	10	73,202	73,211
31	Mononucleotide	(T)12	12	74,048	74,059
32	Mononucleotide	(A)10	10	76,601	76,610
33	Mononucleotide	(T)10	10	80,640	80,649
34	Mononucleotide	(A)11	11	83,558	83,568
35	Mononucleotide	(T)14	14	86,668	86,681
36	Mononucleotide	(A)14	14	109,801	109,814
37	Mononucleotide	(A)10	10	112,073	112,082
38	Mononucleotide	(A)10	10	122,488	122,497
39	Mononucleotide	(T)14	14	132,731	132,744
40	Mononucleotide	(A)14	14	155,864	155,877

**FIGURE 5:**
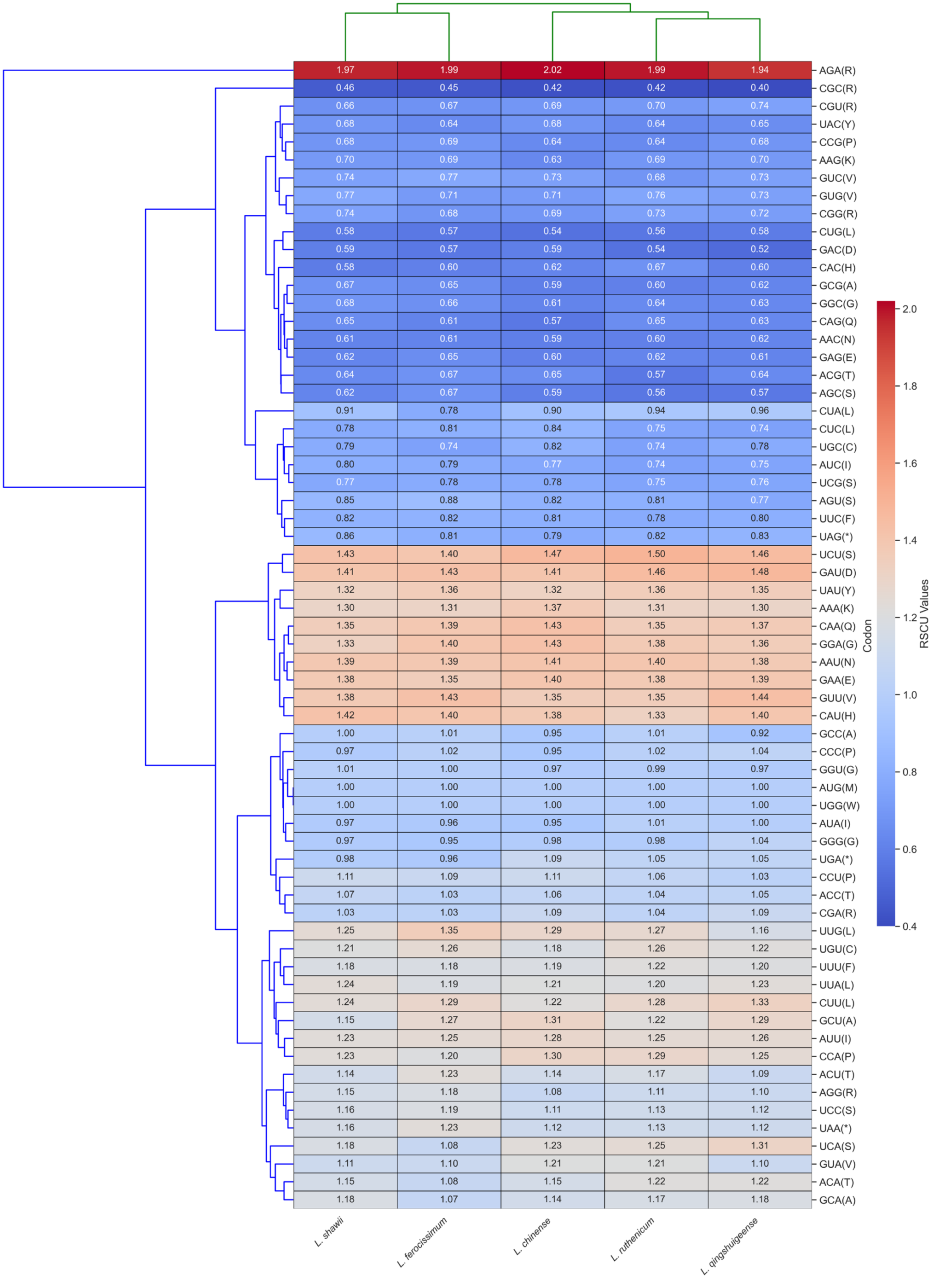
Relative synonymous codon usage and codon preference of *L. shawii* and other closely related taxa.

RSCU analysis revealed a marked preference for A‐ and U‐ending codons in the plastomes of *L. shawii* and its close relatives, that is, *L. ferocissimum* , *L. chinense* , *L. ruthenicum*, and *L. qingshuigeense* (Figure 5). In *L. shawii* , 31 codons exhibited RSCU values higher than 1, indicating frequent and preferential usage of these synonymous codons, while three codons (AUG, GCC, and UGG) had RSCU values equal to 1, reflecting unbiased usage. The remaining 30 codons showed RSCU values below 1, indicating reduced usage and a clear codon usage bias. A similar trend was observed in the related species.

### 3.3 RNA‐Editing Sites Analysis

**FIGURE 6:**
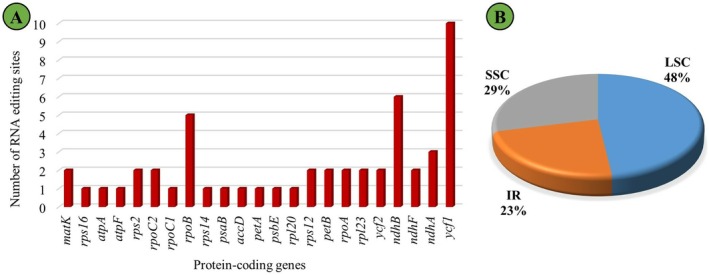
Distribution of RNA editing sites in the Cp genome of *L. shawii* . (A) Number of RNA editing sites of the protein‐coding genes and (B) distribution of the RNA editing sites in SSC, LSC, and IR compartments.

The plastome of *L. shawii* revealed a total of 52 editing sites distributed across different genomic regions (Figure 6). The majority of these sites were located in the LSC region, accounting for 48% of the total, followed by 29% in the SSC region and 23% in the IRs. The *ycf1 gene* in the SSC exhibited the highest number of RNA‐editing events, with 10 sites, indicating a notable post‐transcriptional modification. The *ndhA* and *ndhF* genes in the SSC also showed multiple editing sites, with three and two, respectively. Within the IR regions, *ndhB* was the most extensively edited gene, with six sites, while *rps12*, *rpl23*, and *ycf2* each harbored two editing sites. The LSC region featured a broader array of genes with editing activity: *rpoB* showed the highest number (five sites), followed by *matK*, *rps2*, *rpoC2*, *petB*, and *rpoA*, each with two editing sites. Several other genes, including *rps16*, *atpA*, *atpF*, *rpoC1*, *rps14*, *psaB*, *accD*, *petA*, *psbE*, and *rpl20*, exhibited single editing events.

### 3.4 Expansion‐Contraction of IR

**FIGURE 7:**
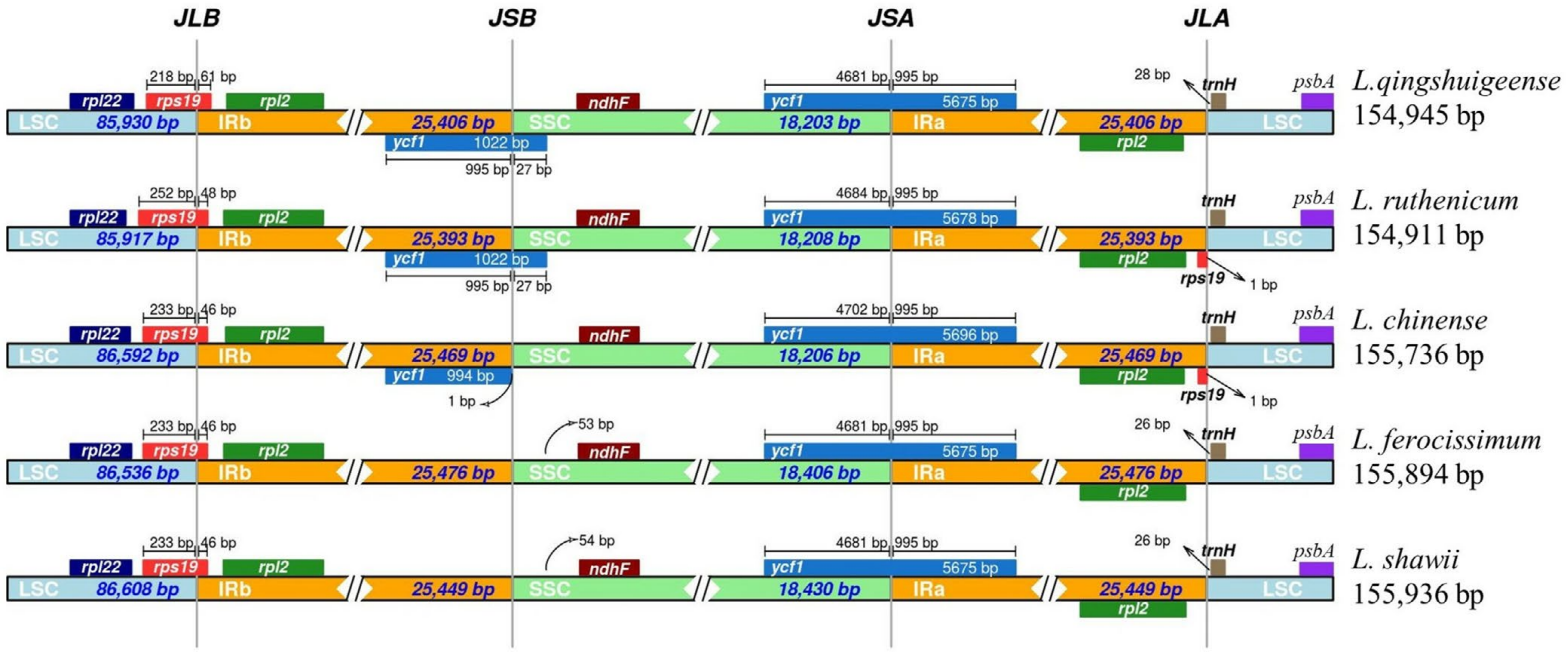
IR expansion and contraction analysis of *L. shawii* plastome within the tribe Lycieae.

The structural organization of the quadripartite plastome, particularly the junction sites, was elucidated through comparative analysis of IR expansion and contraction among *L. shawii* and its closely related species. All examined taxa exhibited highly similar lengths in their LSC, SSC, and IR regions, with the LSC ranging from 85,917 to 86,608 bp and the SSC spanning 18,203 to 18,430 bp. In *L. shawii* , the IR regions showed noticeable expansion compared to *L. qingshuigeense* and *L. ruthenicum*, while a relative contraction was observed in comparison with *L. chinense* and *L. ferocissimum* (Figure 7). Gene positioning at the junction boundaries revealed conserved patterns across all taxa. The *trnH* gene was consistently positioned near the LSC/IRa boundary, while *rps19* marked the LSC/IRb junction. The gene *ycf1*, a conserved open reading frame, spanned the SSC/IRa border in all members, underscoring its structural stability. Similarly, the *rpl2* gene was consistently positioned within the IRb region, and *ndhF* remained in the SSC across all examined plastomes. Collectively, these findings indicate a largely conserved quadripartite structure, with only minor lineage‐specific shifts in IR boundaries among *Lycium* species.

### 3.5 Plastome Divergence and Nucleotide Diversity

**FIGURE 8:**
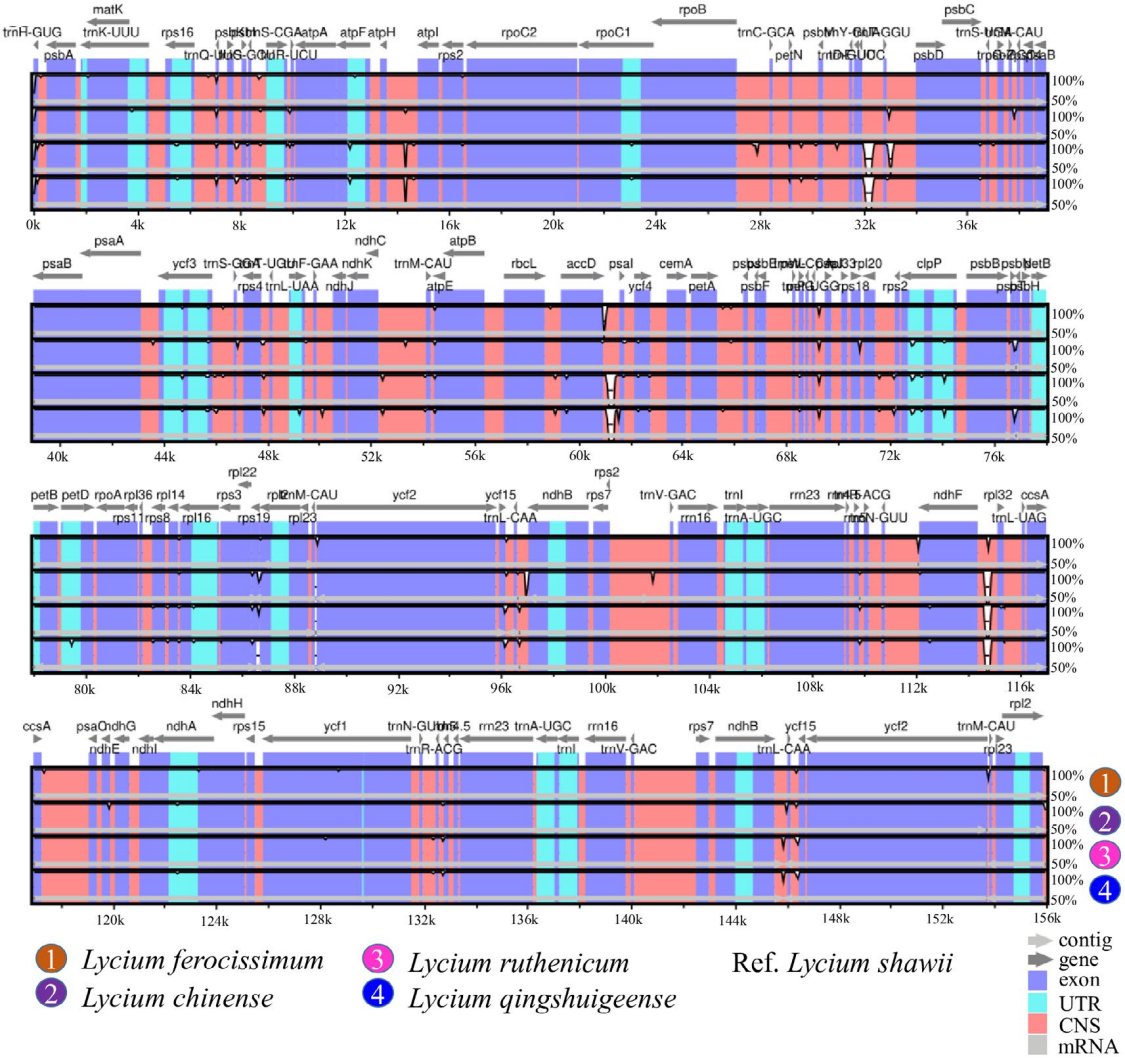
Assessment of genome divergence by aligning the complete Cp genomes of several *Lycium* species with that of *L. shawii* .

Genome divergence analysis based on the mVISTA server indicated that the IRa and IRb regions of *L. shawii* exhibited lower levels of genomic divergence compared with the LSC and SSC. Coding regions were more conserved, whereas non‐coding regions showed greater sequence variability (Figure 8).

**FIGURE 9:**
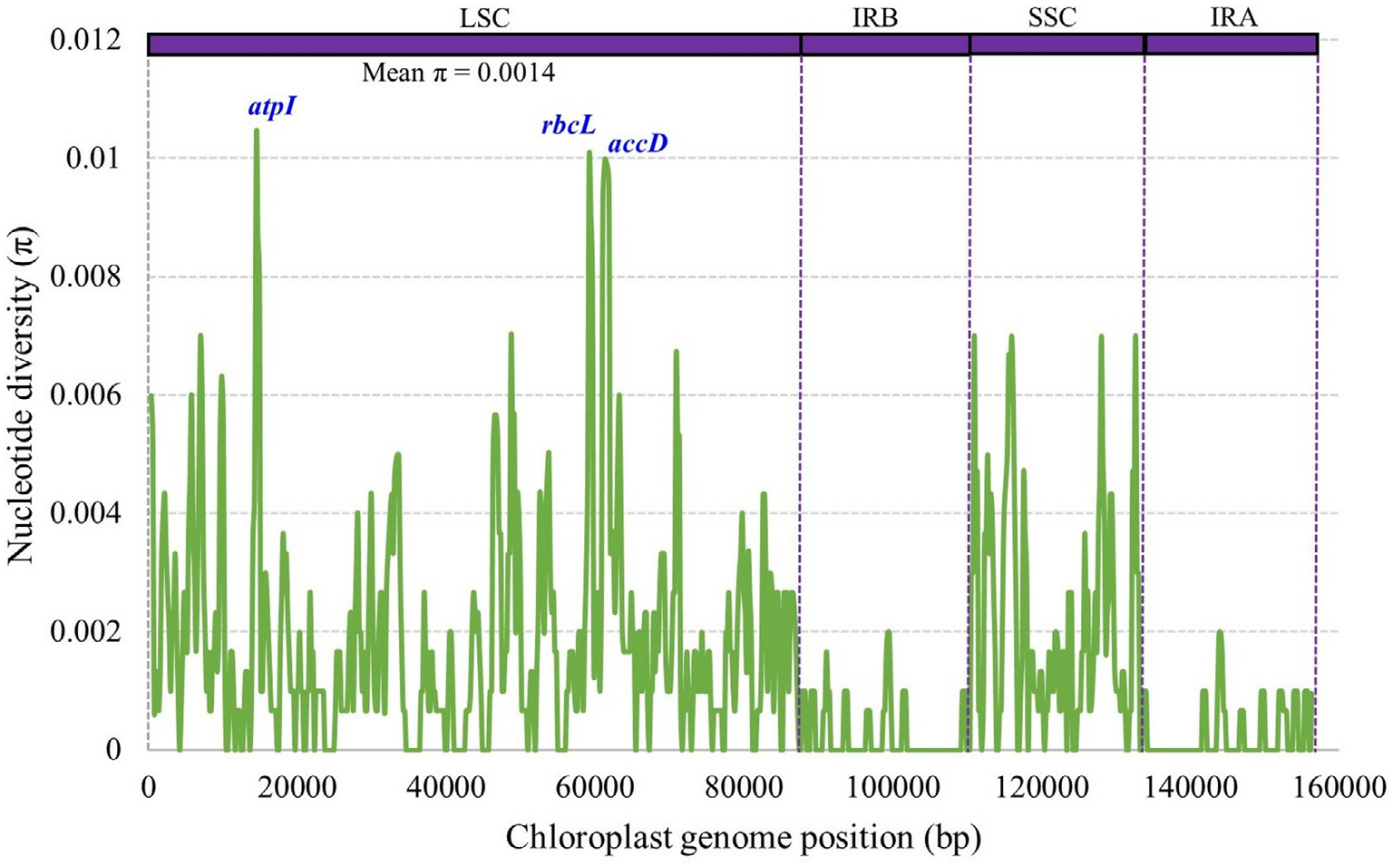
Nucleotide diversity analysis within the Lycieae tribe elucidating hypervariable barcodes in *L. shawii* and closely related taxa.

Nucleotide diversity (*π*) analysis across the complete plastome of *L. shawii* revealed several hypervariable loci that may serve as potential molecular barcodes. The overall mean π value was 0.0014, indicating relatively low sequence divergence at the genome‐wide level (Figure 9). Notably, three genes within the LSC region—*atpI*, *rbcL*, and *accD*—emerged as highly variable, with π values of 0.0103, 0.0100, and 0.0096, respectively, suggesting their potential utility for species delimitation and phylogenetic inference. Comparison among plastome regions showed that the SSC region exhibited higher average nucleotide diversity than the IR regions, consistent with the conserved nature of the IRs. Both IRa and IRb displayed nearly identical diversity patterns, further reinforcing their structural and evolutionary stability.

### 3.6 Molecular Phylogenetics

**FIGURE 10:**
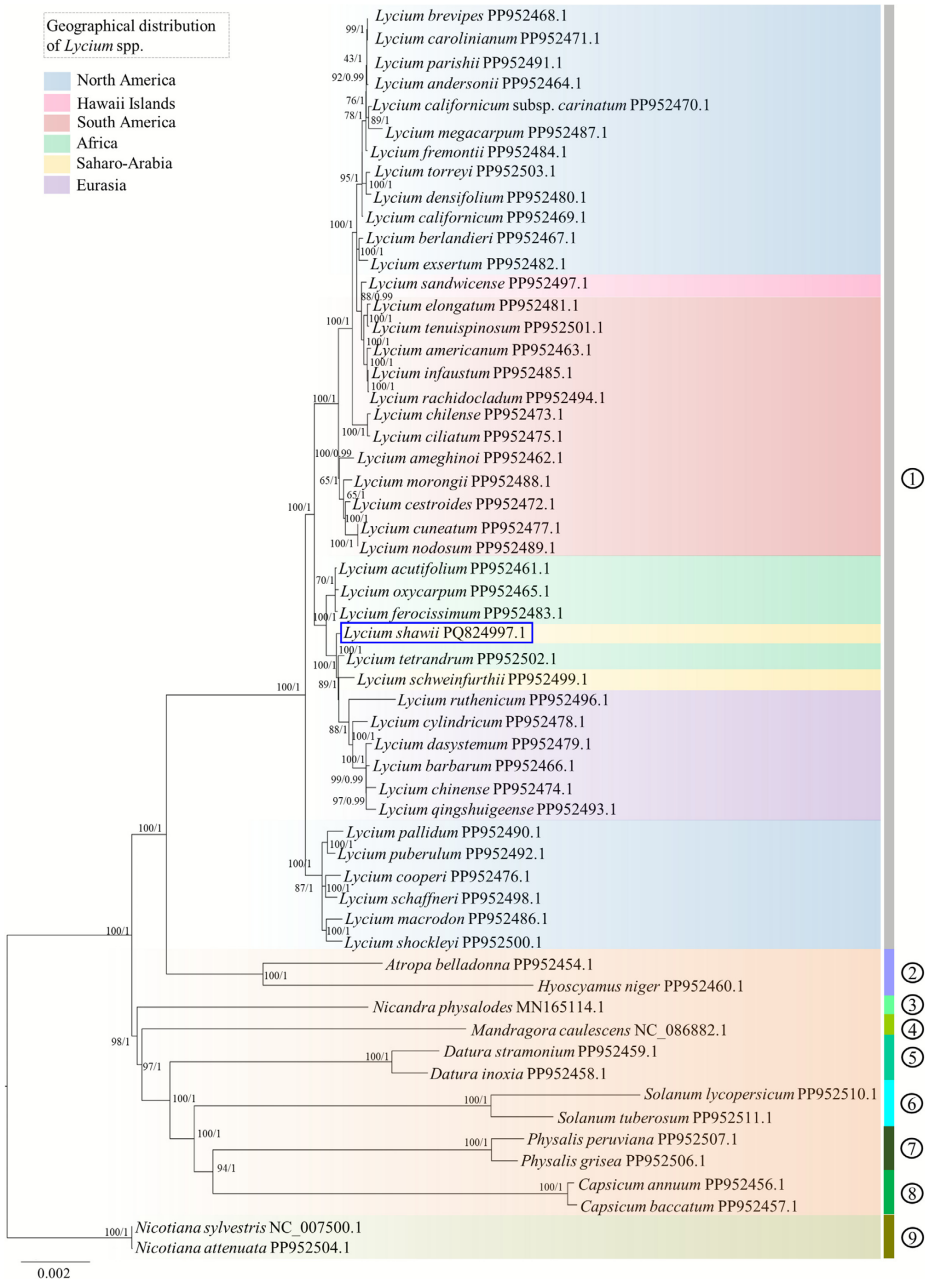
Phylogenetic relationships of *L. shawii* within the subfamily Solanoideae inferred using maximum likelihood (ML) and Bayesian inference (BI) analyses, with representatives of Nicotianoideae used as outgroups. Nodal support values are indicated as ML bootstrap percentages followed by BI posterior probabilities (ML/BI). Numbered circles denote tribal affiliations within Solanoideae: (1) Lycieae, (2) Hyoscyameae, (3) Nicandreae, (4) Mandragoreae, (5) Datureae, (6) Solaneae, (7) Physaleae, and (8) Capsiceae.

Phylogenetic reconstruction using both maximum likelihood (ML) and Bayesian inference (BI) approaches, based on 43 *Lycium* taxa representing six geographic regions, revealed the monophyly of the genus *Lycium* (Figure 10). The ML and BI trees exhibited highly consistent topologies, with most major clades receiving strong bootstrap support and high posterior probabilities, indicating robust resolution of phylogenetic relationships. Within the genus, *L. shawii* was closely associated with the African lineage, suggesting a historical connection or dispersal between the Saharo‐Arabian and African regions. The Saharo‐Arabian taxa, comprising *L. shawii* and *L. schweinfurthii*, were paraphyletic, as African species were nested within their lineage. In contrast, the Eurasian clade, consisting of six species, formed a well‐supported monophyletic group, reflecting a single regional origin. North and South American taxa displayed polyphyletic distributions, occurring in multiple independent lineages across the phylogeny. Notably, a monophyletic cluster of six North American species was recovered as the earliest diverging branch, whereas other North American taxa were interspersed with South American and Hawaiian species, indicating multiple colonization events. Similarly, South American species were distributed across three distinct lineages, some showing close affinities with North American or Hawaiian taxa, reflecting complex biogeographic histories. At higher taxonomic levels, the tribe Lycieae was resolved as closely related to Hyoscyameae. The subfamily Solanoideae, encompassing all included tribes, was recovered as monophyletic and well‐rooted relative to Nicotianoideae, supporting the broader phylogenetic framework of Solanaceae.

### 3.7 Molecular Dating

**FIGURE 11:**
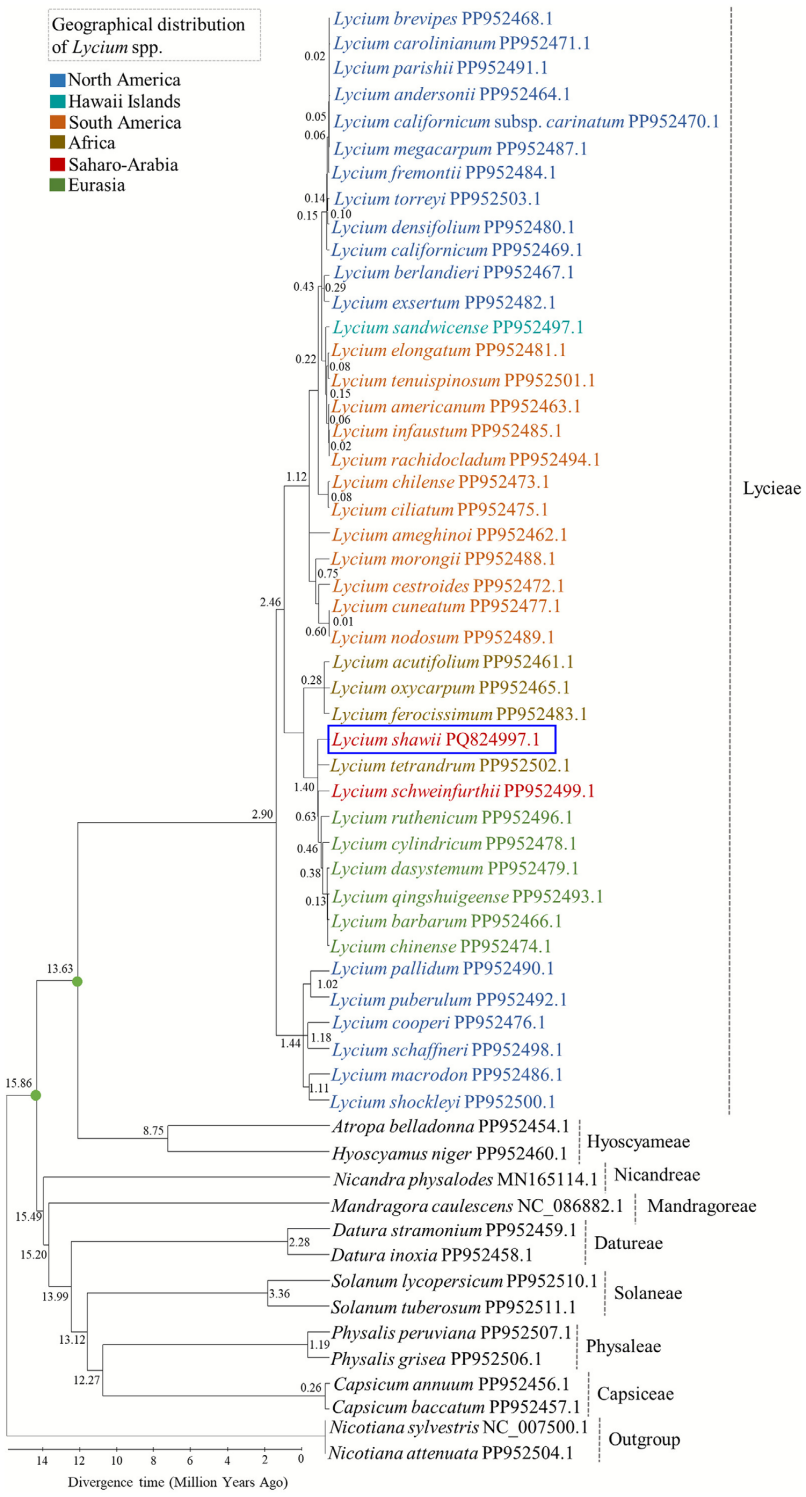
Molecular dating analysis showing the divergence time of *L. shawii* and other closely related taxa within the subfamily Solanoideae.

Molecular dating analysis indicated that the subfamily Solanoideae diverged around 15.86 MYA (million years ago), corresponding to the Langhian age of the Neogene period in the Cenozoic era (Figure 11). Within Solanoideae, the *Lycium* clade diverged approximately 13.63 MYA during the Serravallian age, indicating an early diversification of the genus relative to other lineages within the subfamily. Within this clade, *L. shawii* diverged more recently, approximately 1.40 MYA, during the Calabrian stage of the Early Pleistocene, suggesting that it represents a comparatively young lineage that may have evolved in response to environmental changes in arid and semi‐arid regions.

## 4 Discussion

The genomic structure and gene content of *L. shawii* are largely consistent with those observed in other species, indicating a conserved plastome organization across the lineage. For instance, *L. ruthenicum* was previously reported to possess a chloroplast genome of 155,756 bp, encoding 131 genes, including 86 PCGs, 37 tRNAs, and eight rRNAs (Wang et al. 2019). These similarities reinforce the taxonomic coherence of the genus and validate the accuracy of the annotation and assembly. The nucleotide composition of *L. shawii* was similar to that of the *L. chinense* plastome reported by Yang et al. (2019), reflecting a conserved base composition pattern within *Lycium* (Table 1). Both species exhibited AT‐rich plastomes, with *L. shawii* showing an overall AT content of 62.15%, closely aligning with the 62.2% observed in *L. chinense* . Regionally, the SSC region was the most AT‐rich in both species, with 67.65% in *L. shawii* and 67.60% in *L. chinense* , followed by the LSC region, which recorded 64.10% AT content in both species. The IRs displayed higher GC content in both species, with *L. shawii* exhibiting 43.17% GC and *L. chinense* showing a similar pattern at approximately 43.10%. These closely aligned values highlight the structural and compositional stability of chloroplast genomes across *Lycium* species, supporting their phylogenetic relatedness and evolutionary conservation. The higher GC content in the IRs is particularly significant as it enhances structural stability and replication fidelity. GC base pairs form three hydrogen bonds, whereas AT pairs form only two, resulting in greater thermal stability in GC‐rich regions. This enhanced stability contributes to preserving the conserved architecture of crucial genes, including rRNAs and certain tRNAs, which are typically located within the IR regions. Additionally, higher GC content is associated with reduced mutation rates, helping to maintain essential genetic functions over evolutionary timescales (Ahmed and Rahman 2025).

Per‐base quality profiles demonstrated consistently high sequencing accuracy for both forward and reverse reads. Although the mean Phred score was slightly lower than the quartile values, this reflects the contribution of a small fraction of lower‐quality bases rather than widespread quality deterioration. Quartile scores remained at the maximum reported value (Phred 40) across nearly all positions, indicating highly uniform base calling. A minor decline was observed toward the 3′ end of the reverse reads; however, this decrease was limited and did not compromise overall read integrity, confirming that the data are suitable for reliable genome assembly and downstream analyses.

The exon–intron distribution of the *L. shawii* plastome was found to be largely consistent with that of *L. ruthenicum*, reflecting a high degree of genomic conservation within *Lycium*. In *L. shawii* , 72 out of 84 protein‐coding genes (PCGs) lacked introns, closely resembling the 74 intronless genes reported in *L. ruthenicum* out of 86 PCGs (Wang et al. 2019). In both species, the *ycf3* and *clpP* genes each harbored two introns and three exons, a feature commonly observed in many angiosperm plastomes. In *L. shawii* , most intron‐containing genes were located in the LSC, followed by the IR and SSC regions (Table 3). Additionally, four tRNA genes, such as *trnK‐UUU*, *trnS‐CGA*, *trnL‐UAA*, and *trnA‐UGC*, contained single introns, three of which (*trnK‐UUU*, *trnL‐UAA*, and *trnA‐UGC*) were also present in *L. ruthenicum*. However, *L. ruthenicum* possessed two additional intron‐containing tRNAs (*trnI‐GAU* and *trnV‐UAC*) and an intron‐bearing *rps1* gene, which were not detected in *L. shawii* . These minor variations in intron content and distribution may be attributed to lineage‐specific structural variation, whereas the overall similarities reinforce the conserved exon–intron architecture of *Lycium* plastomes.

The study identified 40 SSRs and 50 longer repeats in *L. shawii* . The overall distribution and abundance of these repeats were consistent with those reported in *L. barbarum* , suggesting conserved patterns of repetitive elements within the genus (Cui et al. 2019). In *L. shawii* , SSRs were predominantly composed of mononucleotide repeats (38 out of 40) (Figure 4), closely aligning with *L. barbarum* , in which 35 of 58 identified SSRs were mononucleotides. While *L. barbarum* contained a higher total number of SSRs (58), the dominance of A/T‐rich mononucleotides in both species reflects the AT‐rich nature of plastomes. Regarding longer repeat structures, *L. shawii* exhibited 23 forward, 19 palindromic, and eight reverse repeats, whereas *L. barbarum* showed a similar pattern with 24 forward, 24 palindromic, and two reverse repeats. Despite slight quantitative differences, the overall repeat profiles indicate a high degree of structural conservation. The identification of SSRs in the *L. shawii* plastome holds significant value for molecular taxonomy and genetic diversity assessments. SSRs, particularly the mono‐, di‐, and tri‐nucleotide motifs detected in this study, may serve as highly informative molecular markers due to their abundance, variability, and co‐dominant inheritance. These markers can facilitate species identification, population structure analysis, and phylogeographic studies within *Lycium* and related taxa (Ahmed and Rahman 2024).

Identifying RNA modification loci in plastomes is crucial for understanding post‐transcriptional modifications that can influence gene expression and protein function. These editing sites, typically involving C‐to‐U conversions, play a key role in restoring conserved amino acids, correcting coding sequences, and ensuring the proper functionality of chloroplast‐encoded proteins (Gao et al. 2023). They are particularly important for regulating plastid gene expression under varying physiological and environmental conditions. In the present study, RNA editing sites were identified in 24 PCGs of *L. shawii* (Figure 6), whereas a higher number (35 PCGs) was reported in *L. barbarum* (Cui et al. 2019). The greater number of edited genes in *L. barbarum* may be attributed to differences in genome annotation protocols, sequence data depth or quality, or the sensitivity of the prediction tools employed. Additionally, minor variation in gene content or structure, such as the presence of pseudogenes or lineage‐specific duplications, may have contributed to a higher count of editing‐affected PCGs in *L. barbarum* .

IR expansion–contraction analysis provides insights into plastome size variation, structural evolution, and gene boundary shifts among related taxa. These dynamics often influence gene content at the IR/SC junctions and serve as valuable markers for phylogenetic and evolutionary studies (Zhu et al. 2016). The IR boundary analysis of *L. shawii* and four related taxa ( *L. chinense* , *L. ruthenicum*, *L. ferocissimum* , and *L. qingshuigeense*) revealed conserved positioning of key genes at junctions: *rps19* at the LSC/IRb border, *trnH* near the IRa/LSC junction, *ycf1* spanning the SSC/IRa boundary, and *ndhF* adjacent to the SSC/IRb border (Figure 7). These patterns are consistent with previous reports for *L. barbarum* , *L. chinense* , *L. ruthenicum*, and *Nicotiana tabacum* (Cui et al. 2019), indicating a conserved IR junction architecture across *Lycium* species and related members of Solanaceae.

Nucleotide diversity (*π*) analysis is a key tool in comparative plastomics for detecting genetic variation across chloroplast genomes and identifying hypervariable regions that serve as potential DNA barcodes (Wang et al. 2019). These regions are valuable for species identification, resolving phylogenetic relationships, and understanding evolutionary patterns in closely related taxa. In this study, *atpI*, *rbcL*, and *accD* located in the LSC region of *L. shawii* and its relatives were identified as hypervariable barcodes (Figure 9). These findings are consistent with previous reports identifying *atpI* and *accD* as hypervariable loci in *L. barbarum* , *L. chinense* , and *L. ruthenicum* (Cui et al. 2019).

The phylogenetic relationships recovered in this study are highly congruent with previous plastid‐based analyses of *Lycium* (Cui et al. 2019; Li et al. 2020; Zhang, Liu, et al. 2024; Zhang, Zhang, et al. 2024) and closely align with the recent comprehensive phylogeny of *Lycium* presented by Yisilam et al. (2025). Both maximum likelihood and Bayesian inference analyses robustly support the monophyly of *Lycium* and reveal pronounced geographic structuring within the genus (Figure 10). Consistent with earlier findings, a well‐supported Eurasian clade was recovered, whereas African and Saharo‐Arabian taxa exhibited paraphyletic relationships, reflecting complex historical connections among these regions. The close affinity of *L. shawii* to African lineages mirrors the placement of Saharo‐Arabian species within African‐Eurasian assemblages reported previously (Yisilam et al. 2025). Furthermore, the early divergence of a monophyletic North American clade, together with the intermixing of remaining North and South American species with Hawaiian taxa, supports scenarios involving multiple dispersal and radiation events, as proposed by Yisilam et al. (2025).

Molecular dating analysis estimated that *L. shawii* originated approximately 1.40 MYA, during the Calabrian stage of the Early Pleistocene epoch of the Cenozoic era (Figure 11). The Calabrian stage (1.80–0.77 MYA), representing the latter half of the Early Pleistocene, was characterized by intensified Northern Hemisphere glaciations and pronounced glacial–interglacial cycles. During this period, the earlier closure of the Central American Seaway continued to influence ocean circulation, strengthening the Gulf Stream and contributing to global cooling. These environmental changes facilitated grassland expansion and promoted the diversification of modern mammalian lineages (Sirenko 2019).

At the genus level, the divergence of the *Lycium* clade was estimated at approximately 13.63 MYA, occurring during the Serravallian stage of the Middle Miocene. This estimate falls between previously reported values. Zhang, Zhang, et al. (2024) inferred an older divergence of 17.7 MYA (Burdigalian age) using complete Cp genomes, whereas Yisilam et al. (2025) estimated a younger age of 10.73 MYA (Tortonian) based on protein‐coding genes (PCGs). These differences likely reflect variation in dataset composition, taxon sampling, and calibration strategies.

The use of complete chloroplast genome sequences provides substantially more phylogenetically informative sites than analyses based solely on PCGs, thereby improving node support, divergence‐time precision, and the reliability of evolutionary inferences (Zhang et al. 2021; Ahmed and Rahman 2024, 2025). In this study, expanded taxon sampling combined with plastome‐wide data yield a refined temporal framework that is consistent with Miocene diversification of *Lycium* and offers a high‐resolution perspective for understanding the evolutionary history of *L. shawii* within Solanoideae.

Although the present study was primarily designed to characterize the chloroplast genome of *L. shawii* , whole‐genome sequencing generated an exceptionally deep dataset (> 400 million paired‐end reads), far exceeding the coverage typically required for plastome assembly. Previous studies have demonstrated that substantially smaller sequencing datasets are sufficient to support the assembly of nuclear and organellar genomes. For example, the nuclear, chloroplast, and mitochondrial genomes of the ornamental palm *Phoenix roebelenii* were successfully assembled using approximately 107 million Illumina reads (Chakravartty and Neelapu 2023). In this context, the extensive sequencing depth obtained for *L. shawii* represents a valuable genomic resource that may facilitate future assembly and characterization of its mitochondrial and nuclear genomes. Although such objectives were beyond the scope of the present chloroplast‐focused investigation, the public availability of the raw sequencing data provides a strong foundation for future studies addressing genome evolution, stress adaptation, and functional genomics within *Lycium* and the broader Solanaceae family (Chakravartty and Neelapu 2024).

## 5 Conclusion

This study presents the complete plastome of *L. shawii* , a desert medicinal species from Saudi Arabia. The plastome exhibits the typical quadripartite structure and contains 128 genes, including 84 protein‐coding genes (PCGs), 36 tRNAs, and eight rRNAs. It also harbors numerous mononucleotide simple sequence repeats (SSRs) as well as longer repeat structures. Comparative genomic analysis confirmed the accuracy of the assembled plastome and highlighted its close affinity with related members of Solanaceae. Three potential hypervariable barcodes—*atpI*, *rbcL*, and *accD*—were identified, providing promising markers for future DNA barcoding and molecular systematics studies. Plastome‐wide phylogenetic analysis corroborated the systematic placement of *L. shawii* within Solanoideae, while molecular dating indicated that the species originated during the Calabrian stage of the Quaternary period in the Cenozoic era. Overall, these findings provide a valuable genomic resource for enhancing taxonomic resolution, guiding conservation strategies, and supporting future molecular studies on this ecologically and medicinally significant species.

## Author Contributions

**Manal Mohammed Ahmed Asiri:** data curation (equal), formal analysis (equal), investigation (lead), methodology (equal), writing – original draft (equal). **Mohammad Ajmal Ali:** conceptualization (lead), formal analysis (equal), funding acquisition (lead), investigation (equal), project administration (equal), resources (equal), supervision (equal), validation (lead), writing – review and editing (equal). **Mona Solaiman Alwahibi:** conceptualization (supporting), formal analysis (equal), methodology (equal), project administration (lead), resources (equal), supervision (equal), visualization (lead), writing – review and editing (equal). **Sheikh Sunzid Ahmed:** data curation (equal), formal analysis (equal), investigation (equal), methodology (equal), validation (equal), visualization (equal), writing – original draft (lead). **M. Oliur Rahman:** investigation (equal), methodology (equal), resources (equal), validation (equal), writing – original draft (supporting), writing – review and editing (lead). **Rajesh Mahato:** data curation (equal), formal analysis (equal), software (lead). **Mohammad Faisal:** data curation (equal), methodology (lead), visualization (supporting). **Soo‐Yong Kim:** formal analysis (equal), investigation (equal), software (equal). **Joongku Lee:** resources (equal), writing – review and editing (equal).

## Conflicts of Interest

The authors declare no conflicts of interest.

## Data Availability

The genome sequence data supporting this study are publicly available in the NCBI GenBank under accession ID PQ824997.1. The associated identifiers are BioProject: PRJNA1203292, SRA: SRR31836791, and BioSample: SAMN45991099.
